# Supplementary material for: Neuroprotective Effects of Krypton Inhalation on Photothrombotic Ischemic Stroke
Source: Biomedicines. 2024 Mar 13;12(3):635. doi: 10.3390/biomedicines12030635 (PMC10968517; doi:10.3390/biomedicines12030635)

Neuroprotective Effects of Krypton Inhalation on Photothrombotic Ischemic Stroke

Viktoriya V. Antonova, Denis N. Silachev, Egor Y. Plotnikov, Irina B. Pevzner, Elmira I. Yakupova, Mikhail V. Pisarev, Ekaterina A. Boeva, Zoya I. Tsokolaeva, Maxim A. Lyubomudrov, Igor V. Shumov, Andrey V. Grechko and Oleg A. Grebenchikov

Supplementary figure S1 of raw uncropped western blot images:

pAKT level in the rat cerebral cortex after krypton exposure.
Raw western blot image to Figure 4a of the main manuscript.

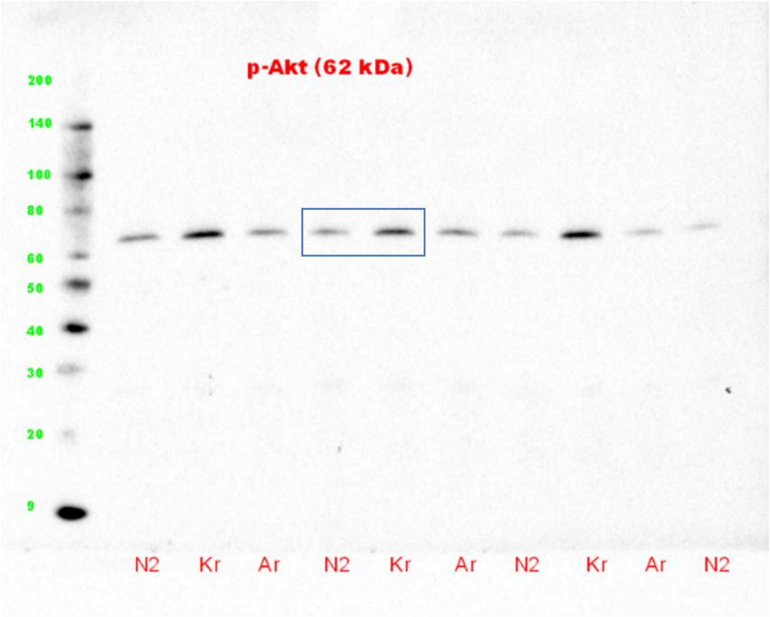

AKT level in the rat cerebral cortex after krypton exposure.
Raw western blot image to Figure 4b of the main manuscript.

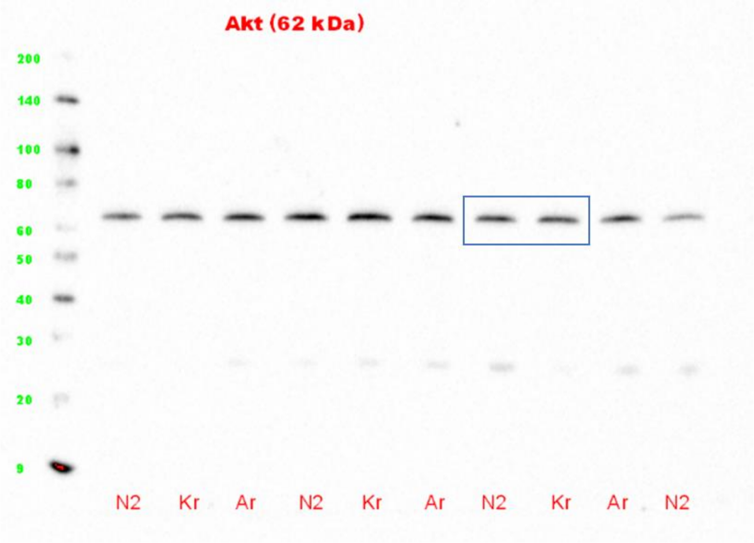

Nrf2 level in the rat cerebral cortex after krypton exposure.
Raw western blot image to Figure 4c of the main manuscript.

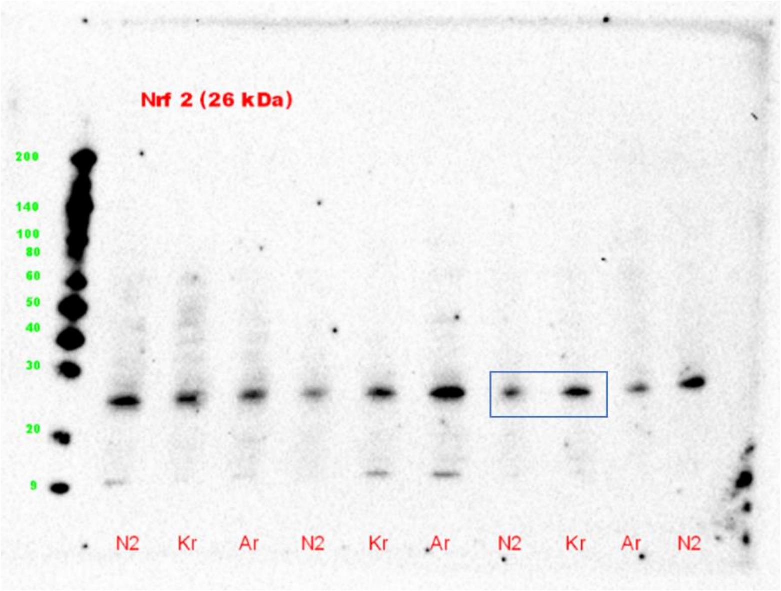

pGSK3b level in the rat cerebral cortex after krypton exposure.
Raw western blot image to Figure 4d of the main manuscript.

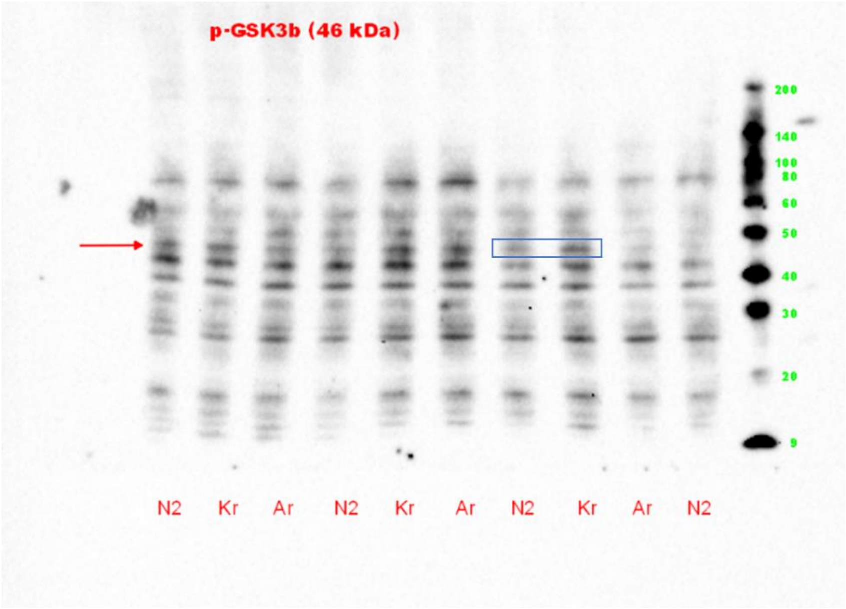

GSK3a/b level in the rat cerebral cortex after krypton exposure.
Raw western blot image to Figure 4e of the main manuscript.

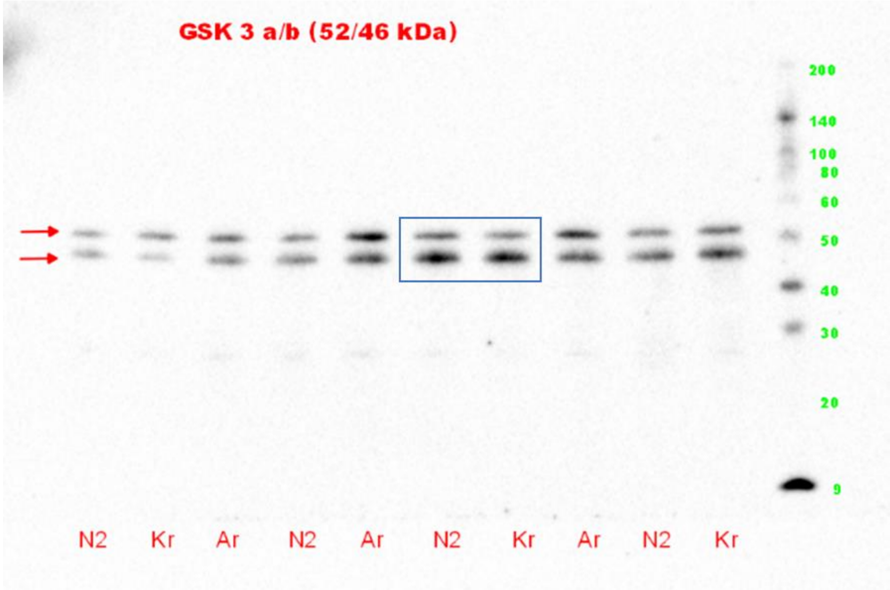

NFkB (p50) level in the rat cerebral cortex after krypton exposure.
Raw western blot image to Figure 4f of the main manuscript.

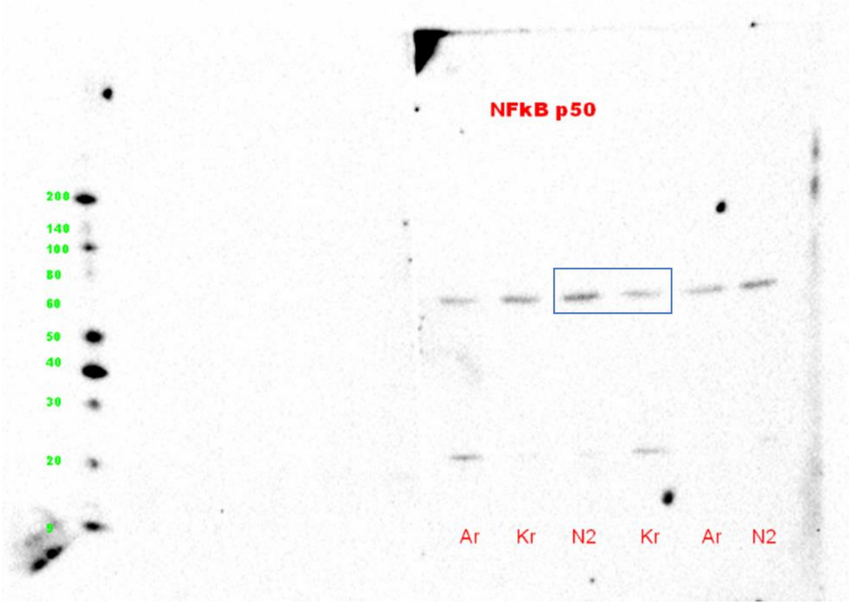

b-actin level in the rat cerebral cortex after krypton exposure as loading control.
Raw western blot image to Figure 4a,b,c,d,f of the main manuscript.

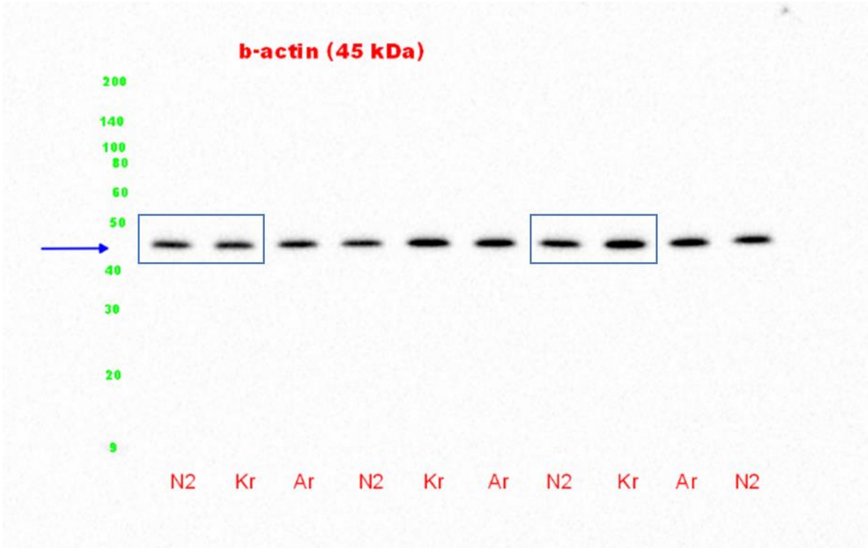

GAPDH level in the rat cerebral cortex after krypton exposure as loading control.
Raw western blot image to Figure 4e of the main manuscript.

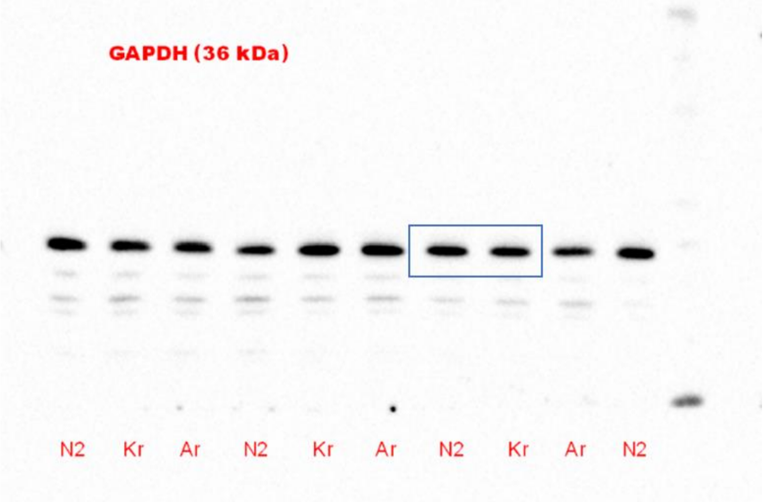

Supplement: Supplementary file 1 [file biomedicines-12-00635-s001.zip › biomedicines-2869486-supplementary.pdf]
